# Parent-reported child appetite moderates relationships between child genetic obesity risk and parental feeding practices

**DOI:** 10.3389/fnut.2023.1174441

**Published:** 2023-05-31

**Authors:** Elena Jansen, Marcus Naymik, Gita Thapaliya, Matt Huentelman, Jennifer Beauchemin, Viren D'Sa, Candace R. Lewis, Sean Deoni, RESONANCE consortium, Sean C. L. Deoni, Susan Carnell

**Affiliations:** ^1^Division of Child and Adolescent Psychiatry, Department of Psychiatry and Behavioral Sciences, Johns Hopkins School of Medicine, Baltimore, MD, United States; ^2^Neurogenomics Division, The Translational Genomics Research Institute (TGen), Phoenix, AZ, United States; ^3^Advanced Baby Imaging Lab, Hasbro Children's Hospital, Rhode Island Hospital, Providence, RI, United States; ^4^School of Life Sciences, Arizona State University, Phoenix, AZ, United States; ^5^Department of Pediatrics, Warren Alpert Medical School at Brown University, Providence, RI, United States; ^6^Maternal, Newborn and Child Health Discovery and Tools, Bill and Melinda Gates Foundation, Seattle, WA, United States

**Keywords:** parental feeding practices, genetic susceptibility to obesity, child eating behavior, Comprehensive Feeding Practices Questionnaire, Child Eating Behavior Questionnaire, child BMI polygenic risk scores

## Abstract

**Background:**

Food parenting practices are associated with child weight. Such associations may reflect the effects of parents' practices on children's food intake and weight. However, longitudinal, qualitative, and behavioral genetic evidence suggests these associations could, in some cases, reflect parents' response to children's genetic risk for obesity, an instance of gene–environment correlation. We tested for gene–environment correlations across multiple domains of food parenting practices and explored the role of parent-reported child appetite in these relationships.

**Materials and methods:**

Data on relevant variables were available for *N* = 197 parent–child dyads (7.54 ± 2.67 years; 44.4% girls) participating in RESONANCE, an ongoing pediatric cohort study. Children's body mass index (BMI) polygenic risk score (PRS) were derived based on adult GWAS data. Parents reported on their feeding practices (Comprehensive Feeding Practices Questionnaire) and their child's eating behavior (Child Eating Behavior Questionnaire). Moderation effects of child eating behaviors on associations between child BMI PRS and parental feeding practices were examined, adjusting for relevant covariates.

**Results:**

Of the 12 parental feeding practices, 2 were associated with child BMI PRS, namely, restriction for weight control (β = 0.182, *p* = 0.011) and teaching about nutrition (β = −0.217, *p* = 0.003). Moderation analyses demonstrated that when children had high genetic obesity risk and showed moderate/high (vs. low) food responsiveness, parents were more likely to restrict food intake to control weight.

**Conclusion:**

Our results indicate that parents may adjust their feeding practices in response to a child's genetic propensity toward higher or lower bodyweight, and the adoption of food restriction to control weight may depend on parental perceptions of the child's appetite. Research using prospective data on child weight and appetite and food parenting from infancy is needed to further investigate how gene–environment relationships evolve through development.

## 1. Introduction

Childhood obesity continues to be a global health problem, with mounting evidence from the United States and other countries suggesting increased weight gain among youth during the recent COVID-19 pandemic (1). Obesity development is driven by individual genetic susceptibility in combination with exposure to an obesogenic environment (2, 3). At the family level, food parenting practices have been posited as determinants of children's eating behaviors and weight (4). However, increasing longitudinal and bidirectional evidence suggests that parents' feeding practices are often influenced by child characteristics, such as appetitive behaviors and bodyweight (5–9), both of which are genetically influenced (10–12). It is, therefore, conceivable that the relationships of food parenting with child appetite and bodyweight, to some degree, reflect an instance of evocative gene–environment correlation (13), such that parents respond to children's genetically influenced obesity risk by modifying their food parenting behaviors.

Two previous studies investigated gene–environment correlation between food parenting practices and children's polygenic risk scores (PRS) for obesity. Importantly, these studies produced contradictory results. Selzam et al. (14), in a sample of 4,445 British 10-year-olds, found that parental restriction of food intake and pressure to eat were associated with child PRS, such that a higher PRS was associated with more restriction and less pressure to eat [both assessed with the Child Feeding Questionnaire (15)]. Guivarch et al. (16) followed 932 French children from age 4 to 24 months and administered five subscales from the Comprehensive Feeding Practices Questionnaire (CFPQ) (17) at the 2-year follow-up to assess food parenting practices in toddlerhood, namely, restriction for health, restriction for weight, pressure to eat, use of food as a reward, and use of food to regulate child emotions. In this study, none of the five subscales was related to the children's obesity PRS, and thus no evidence for a gene–environment correlation was found. Notably, the majority of food parenting practices so far examined in this area of research, and restriction and pressure to eat in particular, are considered to be coercive practices, which consistently show relationships with higher and lower weight, respectively (4). These practices differ from authoritative practices, which include structure and autonomy encouragement, and have been proposed as beneficial for the development of healthy eating patterns (18, 19), particularly in combination with responsive feeding behaviors (20).

To extend the work of these previous studies, we investigated gene–environment correlations between child BMI PRS and parental feeding (a) in children of a wider age range (i.e., from early to later childhood, here 2–15 years of age); (b) across a larger variety of food parenting practices, including structure and autonomy encouragement; and (c) using a potentially more powerful PRS based on the most up-to-date BMI GWAS data and incorporating 2.3+ million single-nucleotide polymorphism (SNPs) that contributed to BMI variation but did not meet the threshold for genome-wide significance. Additionally, to investigate the role of parents' perceptions of children's appetitive behaviors, we tested whether emerging significant relationships between child PRS and parental feeding practices were moderated by parent-reported food responsiveness and satiety responsiveness in children. Based on the previous studies, we hypothesized that restriction and pressure to eat would be associated with the child's BMI PRS. Since the other feeding practices have been less frequently examined, we did not have a specific hypothesis in terms of the direction of effects (e.g., higher obesity risk could elicit more structured feeding or could make it harder to implement structure). We additionally hypothesized that child appetite would moderate the relationships between the child's genetic obesity risk and food parenting practices.

## 2. Materials and methods

### 2.1. Study sample

Data for the present study were obtained from RESONANCE, a large ongoing pediatric cohort study examining early brain development in children (21, 22) with enriched measures of factors relevant to obesity risk (23). RESONANCE forms part of the NIH-funded ECHO program (http://echochildren.org). Details of the study have been published elsewhere (24). In brief, participants were recruited either during pregnancy or when the age of the child was 0–5 years, making use of a variety of methods (e.g., flyers, social media, and in-person events). Study children (*N* = 979, currently active) were followed longitudinally with biannual visits up to the age of 2 years and annual visits beyond that. Children with known risk factors for learning and/or psychiatric disorders were excluded (e.g., birth prior to 32 weeks gestation or birthweight <1,500 g, non-singleton or complicated pregnancy, neurological trauma in the child, and psychiatric history in the parent or sibling) (25). For the current study, participants with relevant data were included, selecting their most recent responses when longitudinal survey data were available. Written consent was obtained from parents or legal guardians in accordance with ethics approval from the host institution's Institutional Review Board.

### 2.2. Measurement tools

#### 2.2.1. Dependent variables

##### 2.2.1.1. Food parenting

Parents completed the Comprehensive Feeding Practices Questionnaire (CFPQ) (17) when children were aged 18 months or older to assess a range of coercive and responsive feeding practices. The 49 items were scored on a 5-point Likert-type scale (1 = never to 5 = always). Questions consisted of statements, such as “How much do you keep track of the high-fat foods that the child eats?” Mean values were created by averaging the respective items contained within each of the 12 subscales. These subscales may be classified into three different domains: (1) structure: Encourage Balance and Variety, Healthy Environment, and Monitoring, (2) autonomy encouragement: Child Control, Involvement, Modeling, and Teaching about Nutrition, and (3) coercion: Emotional Regulation, Food as Reward, Pressure, Restriction for Health, and Restriction for Weight Control.

#### 2.2.2. Independent variable

##### 2.2.2.1. Child BMI polygenic risk scores (PRS)

Saliva was collected from participants using Oragene (DNA Genotek, Ottawa, ON, Canada) saliva collection kits. DNA was extracted with the supplier's isolation kit (DNA Genotek's PT-L2P-5). Sample yield and purity were assessed spectrophotometrically using the NanoDrop ND-1000 (ThermoScientific, Wilmington, DE, USA). Genotyping of the Multi-Ethnic Global Array (MEGA >1.7 million markers) was conducted on an Illumina iScanSystem (Illumina, San Diego, CA, USA). Initial genotype definitions were based on auto-clustering of all samples that had a call rate >0.98 using GenomeStudio (Illumina).

Genotyping data were exported using Genome Studio (Illumina). All the quality control and data filtering steps were conducted using PLINK 1.9 (26). The dataset was filtered by applying the following thresholds: SNP genotyping rate ≥ 95%, minor allele frequency (MAF) ≥ 5%, Hardy–Weinberg equilibrium in unaffected *p* ≥ 1.0 × 10–5, and sample genotyping rate ≥95%. Sex mismatch analysis was conducted using the SNPs located on the × chromosome as implemented in the sex-check function. Sex-discordant samples were removed. Relatedness and duplicated samples were detected by identity by descent (IBD) analysis, estimating the relatedness between all pairs of samples through the calculation of Pi Hat using the genome command. Heterozygosity was computed using the –het function, and samples exceeding a threshold value (defined as ± 3 standard deviations from the study average) were removed. Principal component analysis (PCA) was conducted to detect and remove the outliers. Specifically, we used the identity by similarity (IBS) metric taking into account the first to the tenth closest neighbor and classifying samples with *Z* ≤ −4 as outliers, representing 4 standard deviations below the group mean. VCF files were created and used for imputation via the TOPMed Imputation Server (27–29) with reference panel apps@topmed-r2@1.0.0 (hg38).

Polygenic risk scores for each individual were calculated using a custom R script that multiplied effect allele counts by each allele's corresponding effect size and summed the products as outlined by Marees et al. (30), using the full set of 2.3 million SNPs provided in the GIANT and UK BioBank Meta-analysis GWAS summary statistics for adult BMI (31, 32).

#### 2.2.3. Moderators

##### 2.2.3.1. Child eating behavior

To assess appetitive behaviors in children, the Children's Eating Behavior Questionnaire (CEBQ) (33) was given to parents of children aged 2 years and above. The CEBQ consists of 35 items measured on a Likert-type scale ranging from 1 = never to 5 = always. For the current study, mean values for the subscales food responsiveness (example item “My child is always asking for food”) and satiety responsiveness (example item “My child leaves food on his/her plate at the end of a meal”) were calculated by averaging their respective items and evaluated as moderators reflecting child appetite.

#### 2.2.4. Covariates and demographic information

##### 2.2.4.1. Demographic information

For descriptive purposes only, maternal age, education level, self-reported pre-pregnancy weight, MacArthur Scale of Subjective Social Status (range from 1 to 10 with higher scores indicating higher subjective social status) (34), child race, and ethnicity were used. Child age, sex, and body mass index (BMI) z-scores were included as covariates in the adjusted models. Child weight and height were measured by the study staff during assessments. Child BMI z-scores were calculated using the WHO Anthro version 3.2.2 (35), WHO Anthro Plus, and macros (36). To correct for population stratification, principal components (PCs) were generated using PLINK 1.9 (26) with the default (0.01) minor allele frequency threshold. The top five PCs were selected as covariates based on the visual inspection of the scree plot derived from the accompanying eigenvalues (see Appendix).

### 2.3. Data analysis

Descriptive data for participant characteristics are presented in Table 1. Linear regression analyses were used to examine the relationship between child obesity PRS and food parenting subscales. Models were adjusted for a range of covariates before proceeding to moderation analyses. First, models were adjusted for child age to control for the broad age range in the current sample. Next, models were adjusted by adding sex, then BMI z-score, and finally the five PCs derived from the population stratification analysis. All data analyses were conducted in SPSS 28 software (IBM Corp., Armonk, New York) and the accompanying PROCESS macro for the second analysis step. PROCESS is a path analysis modeling tool that allows the estimation of interactions within moderation models along with simple slopes and regions of significance for probing interactions (37). PROCESS model 1 was selected to test child food responsiveness and satiety responsiveness (M) as respective moderators of the association between the child's BMI PRS (X) and those food parenting practices (Y) that were identified in the first analysis step as significantly associated with PRS. For visualization purposes and to conduct simple slope tests to further validate any moderation effects (*p*-values <0.1), three values of the moderator were created: the mean value (average levels of food/satiety responsiveness), the value that is 1 standard deviation above the mean (high levels of food/satiety responsiveness), and the value that is 1 standard deviation below the mean (low levels of food/satiety responsiveness) (38, 39). Again, child age was included first as a covariate to control for the broad age range. In the second step, moderation models were adjusted for all covariates mentioned earlier. Child BMI PRS and child eating behavior variables were centered to avoid multicollinearity with the interaction term, and bootstrapping procedures were applied (39).

**Table 1:** Sample characteristics.

	***N***	**Mean (or *N*)**	**SD (or %)**	**Range**
**Mothers**				
Maternal age	194	37.66	6.32	23.92–58.25
Maternal education	197			
(Partial) High School		2	1.0	
High School Graduate		19	9.6	
Partial College or Specialized Training		48	24.4	
College graduate		61	31.0	
Graduate training (Masters, PhD)		66	33.5	
MacArthur Scale of Subjective Social Status (34)	174	5.38	1.66	2–9
Maternal pre-pregnancy BMI	176	26.7	6.7	18.3–48.7
**Children**				
Child age (years)	197	7.45	2.75	1.67–15.24
BMIz (WHO reference data)	169	0.37	1.25	−2.90 to 4.10
Wasted		3	1.5	
Healthy weight		115	58.4	
At risk of overweight		32	16.2	
Overweight		15	7.6	
Obesity		4	2.0	
Sex				
Female	197	88	44.7	
Male		109	55.3	
Race	194			
Asian		5	2.5	
Black or African American		9	4.6	
More than 1 race		23	11.6	
White		156	79.2	
Other		1	0.5	
Ethnicity	197			
Hispanic/Latino		31	15.7	
Non-Hispanic/Latino		166	84.3	

## 3. Results

### 3.1. Gene–environment correlations: relationship between child BMI PRS and food parenting subscales

As shown in Table 2, of the 12 feeding practices, 2 were significantly associated with child BMI PRS. Restriction for weight control was positively associated with child BMI PRS, and this relationship remained significant after adjusting for child age, sex, and BMI z-score. However, associations weakened with the addition of five PCs. Teaching about nutrition was negatively associated with child BMI PRS, and this relationship also remained significant after adjusting for child age, sex, and BMI z-score. However, the relationship was not significant when adjusting for all covariates simultaneously.

**Table 2:** Regression analyses examining associations between child BMI PRS and food parenting subscales (CFPQ), adjusted for a range of covariates.

**Regressions**		**Structure**			**Autonomy encouragement**				**Coercion**				
		**Encourage balanced variety**	**Health environment**	**Monitoring**	**Child control**	**Involvement**	**Modeling**	**Teaching nutrition**	**Emotional regulation**	**Food as reward**	**Pressure**	**Restriction for health**	**Restriction for weight control**
Child BMI PRS No covariates	ß	−0.090	−0.040	0.010	−0.096	−0.036	−0.049	**−0.212**	0.075	0.078	0.047	0.033	**0.161**
	*p*-value	0.206	0.581	0.885	0.178	0.619	0.494	**0.003**	0.292	0.278	0.508	0.647	**0.024**
	95% CI	−0.004 0.001	−0.004 0.002	−0.003 0.003	−0.005 0.001	−0.004 0.002	−0.004 0.002	**−0.006** **−0.001**	−0.001 0.003	−0.002 0.005	−0.002 0.005	−0.003 0.005	**0.000 0.005**
	N	197	196	197	197	196	196	**196**	197	196	197	197	**195**
Child BMI PRS With age	ß	−0.102	−0.035	−0.019	−0.111	−0.019	−0.058	**−0.217**	0.047	0.079	0.051	0.032	**0.182**
	*p*-value	0.160	0.632	0.788	0.123	0.792	0.422	**0.003**	0.509	0.275	0.481	0.663	**0.011**
	95% CI	−0.004 0.001	−0.003 0.002	−0.004 0.003	−0.005 0.001	−0.004 0.003	−0.004 0.002	**−0.006** **−0.001**	−0.001 0.003	−0.002 0.006	−0.002 0.005	−0.003 0.005	**0.001 0.006**
	N	197	196	197	197	196	196	**196**	197	196	197	197	**195**
Child BMI PRS With age & sex	ß	−0.106	−0.038	−0.024	−0.106	−0.021	−0.058	**−0.220**	0.057	0.079	0.058	0.035	**0.183**
	*p*-value	0.143	0.604	0.736	0.145	0.777	0.427	**0.002**	0.427	0.280	0.426	0.628	**0.011**
	95% CI	−0.004 0.001	−0.004 0.002	−0.004 0.003	−0.005 0.001	−0.004 0.003	−0.004 0.002	**−0.007** **−0.001**	−0.001 0.003	−0.002 0.006	−0.002 0.005	−0.003 0.005	**0.001 0.006**
	N	197	196	197	197	196	196	**196**	197	196	197	197	**195**
Child BMI PRS With age, sex & BMI z	ß	−0.090	−0.074	0.007	−0.128	−0.030	−0.110	**−0.178**	0.026	0.093	0.136	−0.011	**0.175**
	*p*-value	0.269	0.365	0.929	0.117	0.712	0.185	**0.027**	0.747	0.261	0.093	0.890	**0.028**
	95% CI	−0.004 0.001	−0.005 0.002	−0.003 0.004	−0.006 0.001	−0.004 0.003	−0.006 0.001	**−0.006** **0.000**	−0.002 0.003	−0.002 0.006	−0.001 0.007	−0.005 0.004	**0.000 0.006**
	N	169	168	169	169	168	168	**168**	169	168	169	169	**167**
Child BMI PRS With age, sex, BMI z & five PCs	ß	−0.042	−0.015	−0.020	−0.094	−0.036	−0.058	−0.127	−0.017	0.104	0.033	−0.036	0.074
	*p*-value	0.624	0.862	0.812	0.282	0.670	0.495	0.136	0.943	0.233	0.695	0.670	0.353
	95% CI	−0.003 0.002	−0.004 0.003	−0.004 0.003	−0.005 0.002	−0.005 0.003	−0.005 0.002	−0.005 0.001	−0.003 0.002	−0.002 0.007	−0.003 0.005	−0.006 0.004	−0.001 0.004
	N	169	168	169	169	168	168	168	169	168	169	169	167

The bold values indicate the significant associations at *p*-value <0.05.

### 3.2. Moderation of relationship between child BMI PRS and food parenting by child eating behaviors

Based on the findings of the previous analyses, we conducted moderation analyses with restriction for weight control and teaching about nutrition to test whether observed relationships between child BMI PRS and the two feeding practices were noticed for all levels of child eating behaviors. The results of the moderation analyses are summarized in Table 3. A significant interaction was observed between child BMI PRS and food responsiveness when restriction for weight control was the outcome, and models were either adjusted for child age (*p* = 0.033) or adjusted for child age, sex, BMI z-score, and the five PCs (*p* = 0.028). Satiety responsiveness did not moderate the relationship between child BMI PRS and restriction for weight control in the child age-adjusted model (*p* = 0.054) or the fully adjusted model (*p* = 0.070). No moderation was observed with either child eating behavior when teaching about nutrition was the outcome (all *p's* > 0.050).

**Table 3:** Results of moderation analyses testing child eating behaviors (M) as moderators for the child BMI PRS (X) and parental food parenting (Y) relationship, adjusted for a range of covariates^a^.

		***b***	***SE***	***t***	***p***	**95% CI**	
						**LLCI**	**ULCI**
Model 1a – Restriction for weight control and food responsiveness, adjusted for child age *R*^2^ = 0.1668 *F*(4, 182) = 9.1111, *p* < 0.001							
	Child BMI PRS	0.003	0.001	2.198	0.029	0.001	0.005
	Food responsiveness	0.234	0.054	4.353	<0.001	0.128	0.339
	Child BMI PRS × Food responsiveness	0.003	0.002	2.148	0.033	0.001	0.007
	Age	0.029	0.015	1.914	0.057	−0.001	0.059
Model 1b – Restriction for weight control and food responsiveness, adjusted for all covariates *R*^2^ = 0.2840 *F*(11, 160) = 5.3356, *p* < 0.001							
	Child BMI PRS	0.001	0.001	0.993	0.323	−0.001	0.004
	Food responsiveness	0.171	0.060	2.857	0.005	0.053	0.290
	Child BMI PRS × Food responsiveness	0.004	0.002	2.216	0.028	0.000	0.007
	Age	0.034	0.017	2.050	0.042	0.001	0.067
	Sex	0.034	0.087	0.397	0.692	−0.137	0.206
	BMIz	0.085	0.039	2.187	0.030	0.008	0.161
	PC1	2.216	0.671	3.301	0.001	0.889	3.542
	PC2	0.010	0.636	0.015	0.988	−1.248	1.267
	PC3	0.531	0.847	0.627	0.532	−1.142	2.205
	PC4	−0.596	0.681	−0.875	0.383	−1.943	0.750
	PC5	−0.860	0.665	−1.294	0.198	−2.174	0.454
Model 1c – Restriction for weight control and satiety responsiveness, adjusted for child age *R*^2^ = 0.0655 *F*(4, 182) = 3.187, *p =* 0.0147							
	Child BMI PRS	0.003	0.001	2.398	0.018	0.001	0.005
	Satiety responsiveness	−0.048	0.066	−0.716	0.475	−0.179	0.084
	Child BMI PRS × Satiety responsiveness	−0.004	0.002	−1.944	0.054	−0.008	0.001
	Age	0.028	0.016	1.702	0.090	−0.004	0.060
Model 1d – Restriction for weight control and satiety responsiveness, adjusted for all covariates *R*^2^ = 0.2297 *F*(11, 160) = 4.0120, *p <* 0.0001							
	Child BMI PRS	0.001	0.001	0.793	0.429	−0.002	0.004
	Satiety responsiveness	−0.014	0.073	−0.187	0.852	−0.157	0.130
	Child BMI PRS × Satiety responsiveness	−0.004	0.002	−1.812	0.072	−0.009	0.000
	Age	0.035	0.017	2.031	0.044	0.001	0.070
	Sex	0.016	0.090	0.180	0.858	−0.162	0.194
	BMIz	0.125	0.038	3.277	0.001	0.05	0.200
	PC1	2.320	0.692	3.355	0.001	0.953	3.687
	PC2	0.013	0.665	0.020	0.984	−1.301	1.327
	PC3	0.771	0.878	0.879	0.381	−0.963	2.506
	PC4	−1.041	0.732	−1.421	0.158	−2.488	0.407
	PC5	−1.087	0.687	−1.582	0.116	−2.445	0.271
Model 2a – Teaching about nutrition and food responsiveness, adjusted for child age *R*^2^ = 0.0410 *F*(4, 183) = 1.957, *p* = 0.1029							
	Child BMI PRS	−0.003	0.001	−2.375	0.019	−0.006	−0.001
	Food responsiveness	−0.079	0.063	−1.250	0.213	−0.203	0.046
	Child BMI PRS × Food responsiveness	0.001	0.002	0.281	0.779	−0.003	0.004
	Age	−0.017	0.018	−0.933	0.352	−0.052	0.019
Model 2b – Teaching about nutrition and food responsiveness, adjusted for all covariates *R*^2^ = 0.0946 *F*(11, 161) = 1.4154, *p* = 0.1714							
	Child BMI PRS	−0.001	0.002	−0.631	0.529	−0.004	0.002
	Food responsiveness	0.010	0.074	0.129	0.898	−0.137	0.156
	Child BMI PRS × Food responsiveness	0.002	0.002	0.882	0.379	−0.002	0.006
	Age	−0.004	0.021	−0.181	0.856	−0.045	0.037
	Sex	0.020	0.107	0.185	0.853	−0.192	0.231
	BMIz	−0.089	0.048	−1.858	0.065	−0.183	0.006
	PC1	−1.430	0.83	−1.723	0.087	−3.069	0.210
	PC2	0.108	0.787	0.137	0.892	−1.448	1.663
	PC3	−1.096	1.048	−1.046	0.297	−3.167	0.974
	PC4	−0.406	0.844	−0.482	0.631	−2.074	1.261
	PC5	0.455	0.823	0.553	0.581	−1.172	2.082
Model 2c – Teaching about nutrition and satiety responsiveness, adjusted for child age *R*^2^ = 0.0403 *F*(4, 183) = 1.921, *p* = 0.1087							
	Child BMI PRS	−0.003	0.001	−2.425	0.016	−0.006	−0.001
	Satiety responsiveness	0.082	0.073	1.125	0.262	−0.062	0.227
	Child BMI PRS × Satiety responsiveness	−0.001	0.002	−0.498	0.619	−0.006	0.003
	Age	−0.012	0.018	−0.650	0.517	−0.047	0.024
Model 2d – Teaching about nutrition and satiety responsiveness, adjusted for all covariates *R*^2^ = 0.0965 *F*(11, 161) = 1.4469, *p* = 0.1578							
	Child BMI PRS	−0.001	0.002	−0.650	0.517	−0.005	0.002
	Satiety responsiveness	0.022	0.086	0.256	0.799	−0.148	0.192
	Child BMI PRS × Satiety responsiveness	−0.003	0.003	−1.043	0.298	−0.008	0.003
	Age	−0.001	0.021	−0.054	0.957	−0.042	0.040
	Sex	0.011	0.107	0.106	0.916	−0.200	0.223
	BMIz	−0.084	0.045	−1.863	0.064	−0.174	0.005
	PC1	−1.363	0.824	−1.655	0.100	−2.990	0.265
	PC2	0.065	0.791	0.082	0.935	−1.498	1.628
	PC3	−1.050	1.046	−1.004	0.317	−3.118	1.017
	PC4	−0.601	0.872	−0.689	0.492	−2.325	1.122
	PC5	0.393	0.820	0.479	0.633	−1.227	2.012

^a^Child BMI PRS and child eating behavior variables were mean-centered prior to analysis. *b*, Unstandardized regression coefficient; SE, standard error; LLCI, lower limit confidence interval; ULCI, upper limit confidence interval.

**Figure 1:**
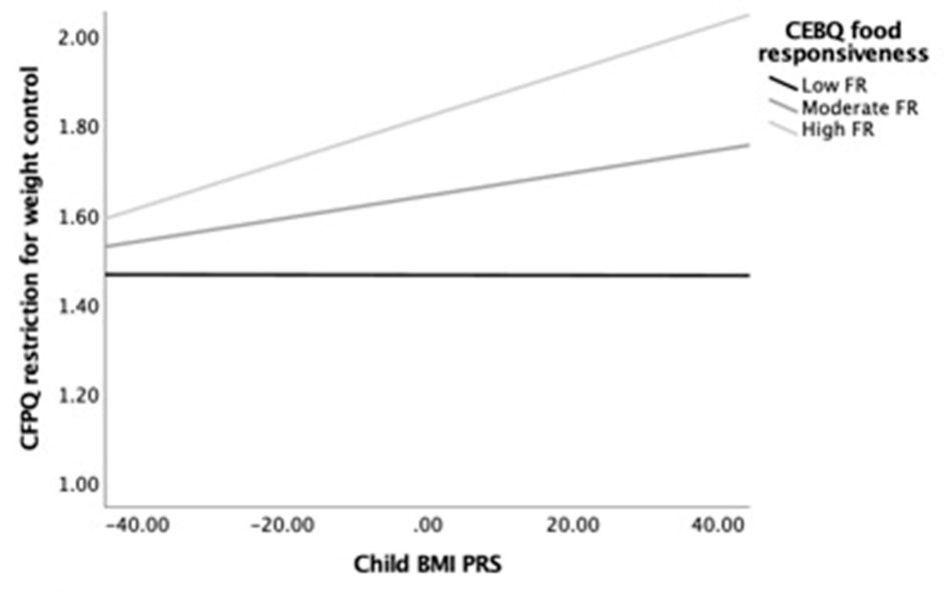
Moderating effects of child BMI PRS (X) on restriction for weight control (Y) among children with low (*M* = 1.53), average (*M* = 2.28), and high (*M* = 3.04) levels of food responsiveness (M), adjusting for child age. At high levels of food responsiveness, the relationship between child BMI PRS and restriction for weight control was strongest (*b* = 0.005, SE_b_ = 0.002, *p* = 0.002, 95% CI = [0.002–0.008]), while at the moderate level, the association was lower but still significant (*b* = 0.003, SE_b_ = 0.001, *p* = 0.029, 95% CI = [0.001–0.005]). In contrast, the association was not significant at low levels of food responsiveness (*b* = 0.000, SE_b_ = 0.002, *p* = 0.988, 95% CI = [−0.003 to 0.003]).

**Figure 2:**
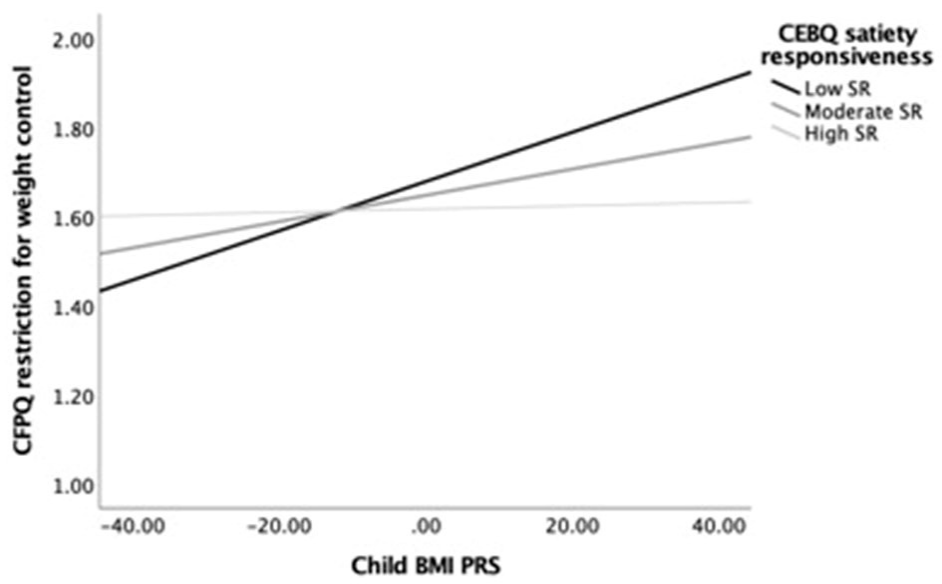
Moderating effects of child BMI PRS (X) on restriction for weight control (Y) among children with low (*M* = 2.26), average (*M* = 2.91), and high (*M* = 3.56) levels of satiety responsiveness (M), adjusting for child age. At low levels of satiety responsiveness, the relationship between child BMI PRS and restriction for weight control was strongest (*b* = 0.006, SE_b_ = 0.002, *p* = 0.002, 95% CI = [0.002–0.009]), while at the moderate level, the association was lower but still significant (*b* = 0.003, SE_b_ = 0.001, *p* = 0.018, 95% CI = [0.001–0.005]). In contrast, the association was not significant at high levels of satiety responsiveness (*b* = 0.001, SE_b_ = 0.002, *p* = 0.841, 95% CI = [−0.003 to 0.004]).

Next, we examined simple slopes for the relationship between child BMI PRS and restriction for weight control by three food responsiveness and three satiety responsiveness groups: low (-1 SD below the mean), moderate/average (the mean), and high (+1 SD above the mean). Notably, in the child age-adjusted model, the gene–environment relationship was significant for children with moderate to high food responsiveness levels but not for those with low levels. In the fully adjusted model, the gene–environment relationship was only significant for children with high food responsiveness levels. Figure 1 provides a visual representation of the child age-adjusted relationships. Similarly, in the child age-adjusted model, the gene–environment relationship was significant for children with low to moderate satiety responsiveness levels but not for those with high levels. In the fully adjusted model, the gene–environment relationship did not reach significance for any level of satiety responsiveness. Figure 2 provides a visual representation of the child age-adjusted relationships.

## 4. Discussion

We examined gene–environment correlations between child BMI PRS and food parenting practices, and tested whether emerging relationships were moderated by parent-reported food responsiveness and satiety responsiveness in children. The results indicate that 2 out of the 12 food parenting practices (i.e., restriction for weight control and teaching about nutrition) are associated with children's genetic risk for obesity. Furthermore, when children have a high genetic obesity risk and show moderate/high (vs. low) food responsiveness, parents are more likely to restrict food intake to control weight.

Our results suggest that parents may adjust their feeding practices in response to a child's genetic propensity toward higher or lower BMI. This effect was evident in our data for the coercive practice of restriction of food intake. Restriction was also found to be associated with children's genetic propensity for obesity in the study by Selzam et al. (14). Our results extend these previous findings by demonstrating that the phenomenon may be specific to restriction for weight control rather than restriction for health reasons, with which no such relationship was found. Further, expanding the focus beyond Selzam et al.'s (14) study, which tested relationships with restriction and pressure only, we found that teaching about nutrition, an autonomy encouragement practice that could promote healthy eating, was also associated with the child's genetic obesity risk, with the negative association suggesting that higher genetic obesity risk is associated with lower teaching about nutrition. Consistent with parenting being a bidirectional process in which parents and children alike exert influence on each other's behavior, these relationships suggest that children's genetics play a role in eliciting specific parental behaviors, as has been established across several domains using twin models (40, 41). While our findings align well with those reported by Selzam et al. (14), they contradict other studies that found no relationships between children's genetic susceptibility to obesity and food parenting practices (16). Incongruent results may arise due to many important differences between studies, including the racial makeup of the sample, the age at which parent and child behaviors are measured, and PRS calculation methods.

To further investigate gene–environment relationships, we tested whether child eating behavior moderated the relationship between child genetics and food parenting. We found that the child's food responsiveness moderated the relationship between child genetics and parental restriction of food intake for weight control reasons, such that when children had a high genetic risk for obesity and showed moderate to high food responsiveness, their parents were more likely to restrict food intake to control weight. In contrast, this relationship was not apparent for children with high genetic risk and low levels of food responsiveness (or low and moderate food responsiveness levels in the fully adjusted model). While the moderation by satiety responsiveness was not statistically significant, the pattern of results in the child age-adjusted models demonstrated that when children have high genetic obesity risk and low or moderate satiety responsiveness, parents may be more likely to restrict food intake to control weight. In contrast, this relationship was not apparent for children with high genetic risk and high levels of satiety responsiveness. Together, our results indicate that when children show levels of appetitive behaviors that are protective against adiposity (42), their high genetic risk for obesity does not elicit certain food parenting practices.

The findings of this study should be interpreted in light of its strengths and limitations. The current study had a smaller sample size than the previous two studies examining gene–environment correlations, which may have limited our ability to detect associations between child BMI PRS and food parenting practices. Particularly, in the fully adjusted models, power may have been reduced due to the small number of participants and the large number of variables in the models. In the current study, we focused on a larger age range in children and also assessed food parenting practices that go beyond coercive practices to expand the field at large. Nonetheless, parental influences may diminish with increasing child age, while genetic influences may strengthen as children grow older [as is found for BMI (43, 44)], thus arguing for the conductance of longitudinal studies that begin in infancy and follow children through later childhood. Additionally, our data did not allow adjustment for parents' own BMI or BMI PRS. Hence, we could not determine to what degree the observed gene–environment relationships were driven by parents' own genetic predisposition toward BMI or other factors, such as their own feeding experience as children (45). We also note that both food parenting practices and child eating behaviors were assessed through parent reports, which are vulnerable to bias. However, the use of a parent-report measure of child appetite also aided in the interpretation of our results, which show that relationships between child genetic obesity risk and food parenting practices depend on parents' *perceptions* of children's eating behaviors. Nevertheless, future studies would benefit from the inclusion of alternative, objective measures or additional raters of eating and feeding behaviors. Finally, since the GWAS used to calculate PRS was derived from adults of European descent, the calculated child BMI PRS may lack validity for the current sample, which included individuals from different genetic populations. Therefore, our results may lack generalizability to children and parents of different ethnic and racial makeup. Given the relevance of culture in eating and mealtime interactions, it is also debatable whether population stratification based on genetic markers, or self-reported race and ethnicity, constitutes more meaningful covariates.

Our findings have implications for the framing of interventions that aim to improve children's eating behavior and prevent obesity. For example, educational interventions for parents, which have shown significant effects on modifying food parenting practices and child eating behavior (46–48), may benefit by acknowledging that parents respond to genetic tendencies demonstrated by their children. However, only a limited number of the measured feeding practices were related to the child's genetic predisposition, and the effects were modest in size. This finding raises the question of whether other factors, such as parents' provision of healthy vs. less healthy foods at home, may be more driven by children's genetic obesity risk. Future studies, including our work with the RESONANCE cohort, should integrate longitudinal child weight data to more effectively investigate interactive and dynamic relationships (49) between children's genetic predispositions, children's appetitive behaviors, parents' feeding practices, and child weight.

## Data availability statement

The original contributions presented in the study are publicly available. This data can be found here: https://doi.org/10.5281/zenodo.7859323.

## Ethics statement

The studies involving human participants were reviewed and approved by Institutional Review Board of Brown University (IRB#: 1500991). The patients/participants provided their written informed consent to participate in this study.

## Author contributions

SC, VD'S, and SD designed the study. JB collected the data. EJ and MN analyzed the data. CRL conducted genomic assays. GT, CRL, and MH contributed to data analysis and interpretation. EJ wrote the first draft of the manuscript. SC, GT, CRL, and MN reviewed and edited the manuscript. All authors approved the final version of the manuscript.

## Members of the RESONANCE consortium include

Sean C. L. Deoni, Bill and Melinda Gates Foundation, Seattle, WA, United States; Viren D'Sa, Department of Pediatrics, Warren Alpert Medical School at Brown University, Providence, RI, United States; Daphne Koinis-Mitchell, Department of Pediatrics, Warren Alpert Medical School at Brown University, Providence, RI, United States; Muriel Bruchhage, Department of Pediatrics, Warren Alpert Medical School at Brown University, Providence, RI, United States; Alexandra Volpe, Department of Pediatrics, Warren Alpert Medical School at Brown University, Providence, RI, United States; Jennifer Beauchemin, Department of Pediatrics, Warren Alpert Medical School at Brown University, Providence, RI, United States; Caroline Wallace, Department of Pediatrics, Warren Alpert Medical School at Brown University, Providence, RI, United States; John Rogers, Department of Pediatrics, Warren Alpert Medical School at Brown University, Providence, RI, United States; Rosa Cano, Department of Pediatrics, Warren Alpert Medical School at Brown University, Providence, RI, United States; Jessica Fernandes, Department of Pediatrics, Warren Alpert Medical School at Brown University, Providence, RI, United States; Elizabeth Walsh, Department of Pediatrics, Warren Alpert Medical School at Brown University, Providence, RI, United States; Brittany Rhodes, Department of Pediatrics, Warren Alpert Medical School at Brown University, Providence, RI, United States; Matthew Huentelman, The Translational Genomics Research Institute, Neurogenomics Division, Phoenix, AZ, United States; Candace Lewis, The Translational Genomics Research Institute, Neurogenomics Division, Phoenix, AZ, United States; Matthew D. De Both, The Translational Genomics Research Institute, Neurogenomics Division, Phoenix, AZ, United States; Marcus A. Naymik, The Translational G-nomics Research Institute, Neurogenomics Division, Phoenix, AZ, United States; Susan Carnell, Department of Psychiatry and Behavioral Sciences, Johns Hopkins University School of Medicine, Baltimore, MD, United States; Elena Jansen, Department of Psychiatry and Behavioral Sciences, Johns Hopkins University School of Medicine, Baltimore, MD, United States; Jennifer R. Sadler, Department of Psychiatry and Behavioral Sciences, Johns Hopkins University School of Medicine, Baltimore, MD, United States; Gita Thapaliya, Department of Psychiatry and Behavioral Sciences, Johns Hopkins University School of Medicine, Baltimore, MD, United States; Vanja Klepac-Ceraj, Department of Biological Sciences, Wellesley College, Wellesley, MA, United States; Kevin Bonham, Department of Biological Sciences, Wellesley College, Wellesley, MA, United States; Monique LeBourgeois, Department of Integrative Physiology, University of Colorado, Boulder, CO, United States; Hans Georg Mueller, Department of Statistics, University of California, Davis, Davis, CA, United States; Jane-Ling Wang, Department of Statistics, University of California, Davis, Davis, CA, United States; Changbo Zhu, Department of Statistics, University of California, Davis, Davis, CA, United States; Yaqing Chen, Department of Statistics, University of California, Davis, Davis, CA, United States; and Joseph Braun, School of Public Health, Brown University, RI, United States.
