# Supplementary material for: Parent-reported child appetite moderates relationships between child genetic obesity risk and parental feeding practices
Source: Front Nutr. 2023 May 31;10:1174441. doi: 10.3389/fnut.2023.1174441 (PMC10266414; doi:10.3389/fnut.2023.1174441)
Supplement: Supplementary file 1 [file Data_Sheet_1.docx]

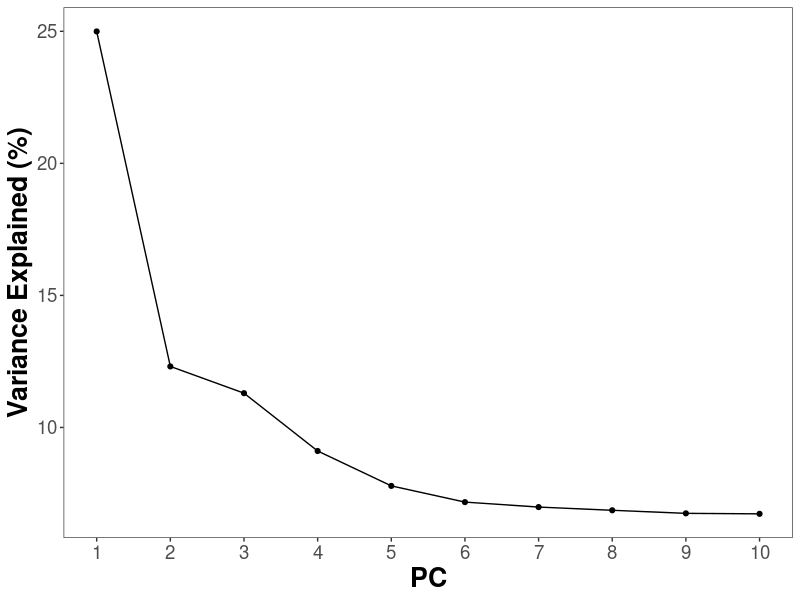


Figure 1: Scree plot to determine the number of Principle components (PC) that will be included in regression and moderation models to correct for population stratification.
